# New virtual case-based assessment method for decision making in undergraduate students: a scale development and validation

**DOI:** 10.1186/1472-6920-13-160

**Published:** 2013-12-03

**Authors:** Zalika Klemenc-Ketis, Janko Kersnik

**Affiliations:** 1 Department of Family Medicine, Maribor Medical School, Taborska ulica 8, 2000 Maribor, Slovenia; 2 Department of Family Medicine, Ljubljana Medical School, Poljanski nasip 58, 1000 Ljubljana, Slovenia

## Abstract

**Background:**

There are many Internet forums where patients can ask medical question and get an answer from doctors. The aim of this study was to develop and validate the rating scale for the assessment of decision-making skills in undergraduate medical students based on such Internet questions.

**Methods:**

This cross-sectional observational study carried out in Medical School of University of Maribor in Slovenia during the family medicine teaching course in the fourth study year. The sample consisted of 159 students. The source of data were the scoring sheets of the students’ reports, assesses by two independent researchers. The assessment tool consisted of 10 items on a five-point Likert scale.

**Results:**

Our final sample consisted of 147 (92.5%) students’ reports. The ICC for matching of the final total scores on assessment tool of both assessors was 0.742. Cronbach’s alpha of the assessment scale was 0.848. Factor analysis revealed four factors: initial assessment, physical examination planning, planning patient management and patient education/involvement.

**Conclusions:**

This assessment tool can be used for assessing undergraduate students’ decision-making based on medical questions asked by real patients in a virtual setting.

## Background

Family practice demands continuous decision-making also in patients with early presentation of undifferentiated and low intensity of symptoms. Traditional medical teaching prepares students for their medical carriers by studying diseases in their full and typical clinical presentations as these represent the majority of hospitalised patients. Family medicine teaching, on the other hand, can offer complementary knowledge, skills and attitudes regarding comprehensive management of patients’ complaints and management of diseases in their early stages often combined with somatoform and medically unexplained symptoms [1,2].

Case-based discussion is a teaching method, which can also stimulate learning of comprehensive patients’ management, patient involvement and information provision [3] and is very useful in family medicine teaching. Because often there are not enough live family practice patients appropriate for covering all educational aspects of medical education, simulated patients or teaching models are not easy available and affordable [4] and it is also difficult to get a comprehensive verbatim transcript of what was asked and said during everyday office consultations, we have to look for alternatives for case-based discussions [3].

Nowadays, more and more teachers use virtual patients or virtual learning modules to stimulate learning of clinical decision-making skills in undergraduate and postgraduate students [5-7]. Namely, virtual patients or e-modules can be easier controlled by teachers and are especially appropriate to enhance their educational value [8,9].

Case-based discussion evolved as a tool in formative assessment in specialty training [10]. Furthermore, virtual patients and e-modules emerged as tools in assessment as well [11]. Previous studies have shown high acceptance of such educational and assessment methods by students and teachers [5,7,12] and have been acknowledged for a good match with classic teaching and assessment methods [6,11]. However, the assessment of decision-making based on virtual patients is poorly studied and the validity and reliability of methods may vary considerably [13,14].

In the era of modern technology, patients use more and more new sources of information, especially Internet [15,16], often in a form of an always open “walk-in” office. Although the quality of such information may be questionable and is difficult to judge its scientific value, it still can be regarded as a valuable source of patient problems which are presented early and in an undefined way which is typical for family medicine. Answering these kinds of virtual patients’ problems presents an additional challenge not only for doctors but also for students, because an important part of consultation, a non-verbal communication, is missing. Also, the students are not able to ask additional questions neither can perform any physical examination. So, one must rely solely on decision-making skills based on history provided by patients in the introductory lines.

Such virtual patients’ problems were the source of patient material for our study.

At the Maribor University, undergraduate study of medicine lasts for six years. Family medicine is taught in the fourth and sixth year of study (seventh and 11^th^ semester) and they are mandatory courses. The curriculum in the seventh semester for family medicine consists of lectures, seminar work and individual student assignments. One of the assignments is also a problem-based learning with virtual clinical case scenarios (see below) [17].

The aim of this study was to develop and test the rating scale for the assessment of decision-making skills in undergraduate medical students.

## Methods

### Study design and settings

This was a cross-sectional observational study carried out in Medical School of University of Maribor in Slovenia during the teaching of family medicine in the fourth study year. The study was approved by the ethics committee of the Department of Family Medicine, Maribor Medical School.

The study followed principles in the Declaration of Helsinki.

### Sample

A sample consisted of all students who attended classes in family medicine in the study years 2009/2010 and 2010/2011 (N = 159). The inclusion criteria were: a student of the fourth-year regularly attending classes in family medicine in the study year 2009/2010 and 2010/2011, regular attendance to problem-based learning with virtual cases exercises and attendance to the final assessment of problem-based learning with virtual cases.

### Problem-based learning with virtual clinical cases

This assignment takes place at the end of the seventh semester and takes three hours. Students get various virtual clinical cases and are asked to write their decisions, counselling, referrals, interventions, etc. The following teaching methods are used: lecture, small group work, one-to-one teaching and discussion. At the end of this exercise, each student is given its own virtual clinical case scenario and has to solve it and write a short report in a predefined format. The general instruction for students is that they have to solve these problems as if a patient would come to their practice with the same question as stated in the virtual case scenario. This report is than assessed by one teacher who gives the student a mark. The aims of these assignments are: teaching of practical aspect of patients’ management in family medicine, teaching of primary care approach to patients, holistic management, comprehensive management, patient involvement strategies and teaching of the approach to patients without clinical examination.

Virtual cases are selected by teachers on the following criteria: new presentation of a problem/symptom, relatively detailed description of the problem and at least some information on the patient, not a clear cut problem, but including some vague or psychological symptomatology, which makes possible a broad spectrum of diagnoses, and demands holistic and comprehensive management of the patient problem(s). Positive side of virtual cases is, that are all already written, written in patient words and do not damage any ethical perspective of breaking confidentiality.

The virtual clinical cases are taken from the freely available e-forum http://med.over.net/forum5/list.php?4. This is a forum on family medicine where questions can be asked by registered users and is moderated by a specialist in family medicine. The teachers themselves choose the appropriate virtual clinical cases and provide them to students.

### Data collection

Source of data for this study were the scoring sheets of the reports of all students in the sample. A tool for scoring (assessment tool) was developed by teachers themselves and based on the two theories of consultation between doctor and patient. The first one was developed by Stott and Davis in 1979 [18] and consists of four parts: management of presenting problems, modification of help-seeking behaviours, management of continuing problems and opportunistic health promotion. The second one was developed by Cohen-Cole and Bird in 1989 [19] and consists of three parts: gathering data to understand the patient’s problems, developing rapport and responding to patient’s emotions and patient education and motivation. We could not chose a single theory to base our assessment tool on due to the need to adapt to the virtual nature of clinical cases, due to the fact that the students were not able to talk to the patients who wrote the clinical questions, and to allow for primary care orientation, comprehensive and holistic management as defined in European definition of general practice.

Each student’s report was independently assessed by two teachers (ZKK and JK) by the assessment tool presented below. Then, the mean value of scores of each item was calculated. The final dataset for statistical analysis therefore consisted of the two teachers’ mean scores for each item.

### Assessment tool

An assessment tool consisted of 10 items: a student 1) asked the appropriate questions regarding patient’s history; 2) proposed the appropriate differential diagnoses; 3) proposed the appropriate clinical examination; 4) proposed the appropriate investigations; 5) proposed the appropriate referrals; 6) proposed the appropriate management; 7) explained the planned investigations and referrals to patient; 8) explained the planned management to patient; 9) explained the probable diagnosis to patient; 10) gave the patient instructions on self-management at home. All items could be graded on a five-point Likert scale: from one (not acceptable) to five (excellent). Maximum total score of the assessment scale was 50 point and minimal total score point was 5 points.

### Data analysis

The data were analysed with the SPSS 13.0 package (SPSS Inc., Chicago, IL). We calculated the descriptive data. For the purpose of determining the level of scores’ matching of both assessors, we calculated the intraclass correlation coefficient (ICC). We calculated the reliability of the assessment tool by Cronbach’s alpha coefficient. We performed factor analysis with principal component analysis extraction method and Quartimax with Keiser normalization rotation method.

## Results

Out of 159 students, 12 did not complete the assignment. So, the final sample consisted of 147 (92.5%) students’ reports. There were 82 (55.8%) reports from the study year 2009/2010 and 65 (44.2%) from the study year 2010/2011. There were 95 (64.6%) female students as authors of the reports.

### Matching of assessment

The ICC for matching of the final total scores on assessment tool of both assessors was 0.742 (Table 1).

**Table 1:** Matching of scores on assessment tool of both assessors

**Item**	**Mean score ± SD (assessor 1)**	**Mean score ± SD (assessor 2)**	**ICC**	**95% lower and higher C. I. for ICC**
Student asked the appropriate questions regarding patient’s history	4.0 ± 0.9	4.2 ± 0.9	0.699	0.583, 0.782
Student proposed the appropriate differential diagnoses	4.0 ± 0.9	4.3 ± 0.8	0.782	0.698, 0.842
Student proposed the appropriate clinical examination	3.6 ± 1.2	3.2 ± 1.6	0.693	0.575, 0.778
Student proposed the appropriate investigations	3.8 ± 1.0	3.9 ± 1.2	0.658	0.526, 0.753
Student proposed the appropriate referrals	3.7 ± 1.1	3.7 ± 1.5	0.665	0.535, 0.758
Student proposed the appropriate management	3.9 ± 1.0	3.9 ± 1.2	0.654	0.521, 0.750
Student explained the planned investigations and referrals to patient	3.2 ± 1.3	3.2 ± 1.5	0.720	0.612, 0.798
Student explained the planned management to patient	3.1 ± 1.3	2.8 ± 1.5	0.729	0.624, 0.804
Student explained the probable diagnosis to patient	2.8 ± 1.2	2.5 ± 1.6	0.617	0.470, 0.724
Student gave the patient instructions on self-management at home	3.1 ± 1.2	3.2 ± 1.5	0.604	0.451, 0.714
Total	35.3 ± 7.9	34.8 ± 7.8	0.742	0.642, 0.814

Legend:

*SD* – standard deviation.

*ICC* – intraclass correlation coefficient.

*C. I.* – confidence interval.

### Reliability

Cronbach’s alpha of the assessment scale was 0.848 (Table 2).

**Table 2:** Assessment tool: item analysis

**Item**	**Corrected item-total correlation**	**Cronbach’s alpha if item deleted**
Student asked the appropriate questions regarding patient’s history	0.590	0.833
Student proposed the appropriate differential diagnoses	0.585	0.833
Student proposed the appropriate clinical examination	0.396	0.850
Student proposed the appropriate investigations	0.525	0.850
Student proposed the appropriate referrals	0.642	0.824
Student proposed the appropriate management	0.590	0.830
Student explained the planned investigations and referrals to patient	0.487	0.840
Student explained the planned management to patient	0.610	0.827
Student explained the probable diagnosis to patient	0.605	0.828
Student gave the patient instructions on self-management at home	0.556	0.833

### Factor analysis

Factor analysis revealed four factors: initial assessment, physical examination planning, planning patient management and patient education/involvement (Table 3). It explained 78.1% variance: factor 1 – planning patient management – explained 28.7%, factor 2 – patient education/involvement – 25.2%, factor 3 – initial assessment – 13.3% and factor 4 – physical examination planning – 10.9% variance.

**Table 3:** Assessment tool: factor analysis*

**Item**	**Factor 1 – planning patient management**	**Factor 2 – patient education/involvement**	**Factor 3 – initial assessment**	**Factor 4 – physical examination planning**
Student asked the appropriate questions regarding patient’s history	0.488	0.171	**0.729**	0.077
Student proposed the appropriate differential diagnoses	0.396	0.276	**0.772**	−0.051
Student proposed the appropriate clinical examination	0.372	0.198	0.006	**0.813**
Student proposed the appropriate investigations	**0.760**	−0.027	0.226	0.290
Student proposed the appropriate referrals	**0.805**	0.194	0.025	0.293
Student proposed the appropriate management	**0.795**	0.161	0.196	−0.153
Student explained the planned investigations and referrals to patient	−0.016	**0.763**	0.296	0.224
Student explained the planned management to patient	0.149	**0.879**	0.107	0.101
Student explained the probable diagnosis to patient	0.317	**0.820**	−0.058	−0.104
Student gave the patient instructions on self-management at home	0.498	**0.528**	−0.064	−0.400

*Rotated component matrix using Quartimax method with Kaiser Normalization.

## Discussion

The tool for the assessment of virtual case-based decision-making showed good psychometric characteristics by good interclass correlations and high Cronbach’s alpha. Factor analysis revealed four factors, which are essential to any consultation: initial assessment, physical examination planning, planning patient management and patient education/involvement.

When developing this assessment toll, we used two consultation theories [18,19]. The factors that emerged from our analysis correlate well with the factors from these two theoretical frameworks and also with other consultation theories (Table 4) [18,20-22]. Therefore, we can claim that our tool is comprehensive and cover all essential parts of consultation in family medicine: establishing of initial contact with patient and developing a range of possible early diagnoses [23], physical examination planning and also very important part of consultation – patient involving in treatment (planning, explaining of further procedures, giving advices on self-management at home, appropriate actions in case of health status deterioration and rational use of health services in future). These consultation parts are a basis of each consultation and also present one of the educational aims of family medicine course [24] and now can be assessed by using this newly developed tool.

**Table 4:** Comparison of different consultation models according to the factors found in the present study

**Tool/model**	**Factor/step in consultation**	**Factor/step in consultation**	**Factor/step in consultation**	**Factor/step in consultation**
Our new assessment tool	Initial assessment	Physical examination planning	Planning patient management	Patient education/involvement
Byrne & Long model [20]	The doctor establishes a relationship with the patient.	The doctor conducts a verbal or physical examination or both.	The doctor, or the doctor and the patient or the patient (in that order of probability) considers the condition.	The doctor, and the patient, detail treatment or further investigation.
	The doctor either attempts to discover or actually discovers the reason for the patient’s attendance.			
Stott & Davis model [18]	Management of Presenting problems.			Opportunistic Health Promotion.
				Modification of Help Seeking Behaviour.
Pendleton model [21]	Reason for Attending		Doctor and patient choose an action for each problem.	Involve patient in management, sharing appropriate responsibility.
			Sharing understanding.	
Calgary-Cambridge model [22]	Initiating the session	Gathering information	Explanation and planning	Explanation and planning
	Gathering information			

Many tools have been developed for the assessment of consultation, for direct observation of clinical skills performance, mainly on the level of specialty training [25]. However, only few of them have been thoroughly evaluated and tested [25]. Also, many of them are focused mainly on the assessment of clinical skills and management of presenting problems. Only few of them included also the assessment of patient involvement in consultation, preventive activities provided by doctors, modification of health-care utilization practices and self-management advices given [25]. These shortcomings have been overcome by our newly developed tool which, in addition, also showed that students performed poorly in some of these tasks.

There have also been several tools for assessment of students developed based on virtual cases or virtual patients [6,7,9,11,14]. A study from USA [11] assessed a class of 155 second year medical students to evaluate problem-based learning using a virtual patient. The results showed good knowledge of students but the study did not evaluate any assessment tool. Another study from Sweden [6] compared the assessment results between virtual patient simulation and regular course exam and found superior results in the group of students with virtual patients. However, the study did not provide any evaluation of the assessment tools. Yet another study from Germany [14] used a case-based online assessment tool by simulating consultations with virtual patients. This tool proved to be both valid and reliable and highly correlated to the results of a written exam. It seems that virtual patients and virtual clinical cases are often used in medical teaching but lack the reliable and valid assessment tools.

This study put forward a new method of teaching and assessing undergraduate students – the medical question asked by real patients in virtual settings without the possibility of further exploration of patients. This medical question was the only peace of information given to students. Their answers were assessed by the newly developed assessment tool. We demonstrated that it can be used for assessing undergraduate students’ decision-making in virtual cases. Based on this tool, students can be reliably assessed by only one assessor. Although not included in our study, this tool could be used when assessing the students’ consultations and decision-making also in real patients as it covers all essential parts of consultation. It could also be used to assess written assignments where students have to report on patient management and/or decision making. In formative assessment, it could also be used for self-assessment of students [5].

The main strength of this study is good matching of the assessment scores of both assessors. Similar studies [26,27] on portfolio assessment also showed good matching between independent assessors and their authors therefore suggested that there is a possibility of reducing the number of assessors while maintaining a sufficient level of reliability. This holds true also for our instrument which can result in an increased feasibility of this newly developed assessment tool for both students and assessors. Another strength is also the fact that the tool is based on consultation theories which gives it additional validity besides good validity and reliability showed in our study.

There are also several limitations of the study. The main limitation is that we did not evaluate the educational value of such virtual cases. The students were not assessed before and after the appropriate education took place. This was due to the inability of the researchers to do so because of the organizational problems in the course. However, the aim of this study was not to test the educational value of this tool but to validate it. Another limitation is also the fact that we did not compare the assessment based on this tool to the classical assessment used in our students.

Further studies should evaluate the educational value of this tool, test it also in real-life clinical cases and compare it to other assessment methods. The tool would be given additional value also by testing it in different learners’ populations (i.e. trainees, continuous medical education) and by testing it among the medical students when they face real patients in FPs’ offices.

## Conclusions

Medical questions by patients on Internet health forums can be used for assessing clinical decision-making in undergraduate medical students. A tool for assessing clinical decision-making in undergraduate students using medical questions on Internet health forums, presented in this paper, is a valid and reliable tool. It also can be used by only one assessor.

## Competing interests

Authors declare they have no competing interests.

## Authors’ contributions

ZKK and JK conceived the study, participated in its design and coordinated it. ZKK performed the statistical analysis. ZKK and JK interpreted the results. ZKK drafted the manuscript and JK approved its final version.

## Pre-publication history

The pre-publication history for this paper can be accessed here:

http://www.biomedcentral.com/1472-6920/13/160/prepub
